# Effect of Glucose Levels on Cardiovascular Risk

**DOI:** 10.3390/cells11193034

**Published:** 2022-09-28

**Authors:** Anastasia V. Poznyak, Larisa Litvinova, Paolo Poggio, Vasily N. Sukhorukov, Alexander N. Orekhov

**Affiliations:** 1 Institute for Atherosclerosis Research, 121609 Moscow, Russia; 2 Center for Immunology and Cellular Biotechnology, Immanuel Kant Baltic Federal University, 236001 Kaliningrad, Russia; 3 Unit for Study of Aortic, Valvular and Coronary Pathologies, Centro Cardiologico Monzino IRCCS, 20138 Milan, Italy; 4 Laboratory of Angiopathology, Institute of General Pathology and Pathophysiology, 125315 Moscow, Russia; 5 Petrovsky National Research Centre of Surgery, 2, 119991 Moscow, Russia

**Keywords:** atherosclerosis, diabetes mellitus, glucose, blood glucose

## Abstract

Cardiovascular diseases remain the leading cause of death and disability. The development of cardiovascular diseases is traditionally associated with various risk factors, most of which are somehow related to an unhealthy lifestyle (smoking, obesity, lack of physical activity, etc.). There are also risk factors associated with genetic predisposition, as well as the presence of concomitant diseases, especially chronic ones. One of the most striking examples is, of course, type 2 diabetes. This metabolic disorder is associated with impaired carbohydrate metabolism. The main clinical manifestation of type 2 diabetes is elevated blood glucose levels. The link between diabetes and CVD is well known, so it is logical to assume that elevated glucose levels may be important, to some extent, in the context of heart and vascular disease. In this review, we tried to summarize data on the possible role of blood glucose as a risk factor for the development of CVD.

## 1. Blood Glucose as a Risk Factor

The identification of a significant, continuous, steady increase in various relatively short-term-adjusted mortality risks, with a rise in fasting blood glucose (BG), starting from the lower limit of the normal range for people who do not suffer from glucose intolerance, who are mainly in the stable chronic phase of cardiovascular disease, suggests that this point of view may need to be revised [1]. There is no evidence of a lower threshold for these patients: adjusted 2-year mortality is 2.42 times higher (7.23%) at 89 [plasma equivalent = 100] mg/dL than at 60 mg/dL (2.99%) [plasma equivalent = 67] and 3.81 times higher (11.38%) at 119 [plasma equivalent = 134] mg/dL than at 60 mg/dL, with the same sharp increase for CVM and non-CVM. Therefore, BG can provide a new and powerful predictor of mortality in these high-risk patients [2].

The results of several studies have revealed that when the glucose level reaches below the critical point of diabetes, it symbolizes an extremely high risk of death for individuals in the acute phase of CAD or in those who undergo percutaneous coronary intervention [3]. The important association between nondiabetic glucose levels and mortality in patients with a stable chronic phase of cardiovascular disease was first highlighted by Fisman et al. In their study, nondiabetic patients with CAD were divided into categories of fasting plasma glucose (b110, 110 to 126, and N126 mg/dL); the results of the study showed that mortality increased significantly in all three categories [4].

Studies conducted later also demonstrated an elevation in mortality in these categories. The outcomes verified and extended earlier conclusions. They confirmed a direct and strong link between mortality and blood glucose in patients in the chronic phase of any form of cardiovascular disease. They also quantitatively expanded the previous results, demonstrating that in addition to the elevation in the three categories mentioned above, there was a continuous, steep-graded association of various mortality risks with blood glucose, which covers the entire normal and sub-diabetic range, without specifying a lower threshold. Amongst other things, there was an elevation in the rate of these risks from the lower to the upper limit of the regular range [1,5].

Since subjects with glucose intolerance (specifically, all patients with diabetes mellitus) were excluded and adjusted for different CV risk factors (such factors as age, sex, BMI, systolic BP, total cholesterol, cigarette smoking, and the use of antihypertensive drugs), the chance that the connection of glucose deaths tracked here is linked with known risk factors is negligible [6]. On the other hand, in a non-experimental study, this possibility can never be completely ruled out. The mechanism(s) by which glucose is able to elevate the risk of mortality in patients with CVD has not yet been studied. Glucose is able to directly harm the endothelial or atherosclerotic plaque of blood vessels, mediated by non-enzymatic glycosylation of low-density lipoprotein (LDL) cholesterol and other apolipoproteins and blood clotting factors [7].

**Figure 1:**
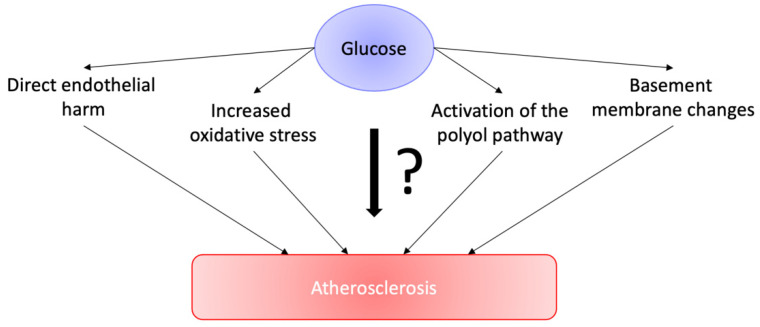
Potential relationship between glucose and atherosclerosis.

There are other mechanisms, due to which high glucose levels can worsen atherosclerosis or heart failure, including elevated oxidative stress, activation of the polyol pathway, and changes in the basement membrane. This potential relationship is represented in Figure 1. Alternatively, insulin resistance may be a factor leading to the aggravation of cardiovascular disease, with increased glucose levels as a sign of insulin resistance. Many studies have demonstrated that regardless of other risk factors, hyperinsulinemia can also be called a sign of CAD in healthy people [8]. Insulin resistance is able to worsen ischemic myocardial damage, provoking a reduction in glucose utilization and an elevation in the utilization of free fatty acids, elevation of oxygen demand, and decreasing contractility. Hyperinsulinemia was linked with a reduction in fibrinolysis, due to an elevation in the level of plasminogen activator inhibitor-1 [9].

Today, we are unable to gain access to the effect of insulin resistance on mortality, since in the Framingham Heart Study, insulin levels were not measured [10]. At this stage, there is not enough data to make a solid analysis of the relationship of various cause-specific CVMs with BG or to study non-CVM causes. Theoretically, the growth of CVMs, not being related to glucose, may be related to mortality from kidney diseases, which, according to the Framingham classification, are not cardiovascular diseases [11].

Prospective studies, which were conducted in the past and aimed to determine an association of non-diabetic glucose levels with mortality, were conducted almost exclusively on healthy individuals and thus, are not directly comparable with the outcomes obtained in individuals with cardiovascular disease. The study’s outcomes revealed mixed connections of mortality with glucose [12].

Most of the studies reported that the increase in risk began only in the upper range of the norm; this increase was about 50%. This differs significantly from the results presented here for cardiovascular patients, in whom the increase in risk begins at the lower part of the normal range and is approximately 242% higher at the upper part of the normal range: BG = 89 [plasma equivalent = 100] mg/dL (normal range approx. from 70 mg/dL (3.9 mmol/L) and 100 mg/dL (5.6 mmol/L)) [13].

## 2. Diabetes Mellitus (DM)

Other risk factors that may stimulate cardiovascular diseases are impaired glucose tolerance and diabetes mellitus, which also correlates with a high risk of CAD. Moreover, patients suffering from DM, who have not previously experienced a myocardial infarction, (MI) have equal risks to an MI, as well as patients without diabetes and previous cases of MI [14].

The Framingham Heart Study (FHS) is a long-term study, which observes different generations in order to determine common factors or features that promote cardiovascular disease. The FHS showed that diabetic patients have a higher incidence of heart failure after adjusting for other risk factors linked with disease (age, BP, weight, and levels of cholesterol) [10]. Therefore, the anomalous structure and performance of the myocardium in people with diabetes mellitus without other risk factors is classified as diabetic cardiomyopathy. Diabetes mellitus is also important in the development of atrial fibrillation (AF), which is the most common persistent arrhythmia globally, and boosts the risk of thromboembolic stroke [15].

Chronic hyperglycemia can lead to the development of complications in DM. Another risk factor for CVD is potentially the variability of glycemia. Frequent and large fluctuations in glucose levels also result in CVD that does not depend on chronic hyperglycemia [16]. 

Epidemiology of Diabetes: Joint Analysis of Diagnostic Criteria in Europe (DECODE) is a study of about 30,000 subjects from 20 European countries, aimed at studying and analyzing the relationship between glucose tolerance and mortality. This study [17,18,19] showed that higher 2-h plasma glucose levels after an oral glucose tolerance test correlated with a rise in CV mortality, and ADVANCE [20] and VADT [21] studies showed that acute hypoglycemia in patients with T2D under intensive control was linked with a higher risk of CVD [22].

Studies showed that the majority of patients with type 1 diabetes experienced severe hypoglycemia because of the insulin, and 42.3% and 51.1% of type 2 diabetic patients were due to sulphonylurea (SU) and insulin, respectively [23]. Investigation by Signorovitch et al. revealed that the use of thiazolidinediones (TZD) (14.5%), sulphonylurea (38.2%), and biguanide (56.3%) were tightly linked to the severe hypoglycemia development [24]. Another study was conducted to find out the frequency of the severe hypoglycemia among new users of insulin and oral anti-diabetic drugs. Moisan et al. used the databases of the Quebec health insurance board and the Quebec registry of hospitalizations between 1 January 2000 and 31 December 2000 to recruit 188,659 new users of anti-diabetic treatment. A total of 3575 (1.9%) individuals had at least one hypoglycemia-related ED visit. This study also showed the greater use of metformin (45.0%), as compared with sulphonylurea (32.1%) [25].

Hsu et al. showed that the number of insulin and sulphonylurea users was significantly greater in patients with severe hypoglycemia (24.2% for insulin, 67.8% for SU) than in patients without hypoglycemia (4.35% and 54.95%, respectively) [26]. According to Ben-Ami et al., the use of glyburide as a mono-therapy (51.5%) and as combination therapy with metformin appeared to be the most wide-spread drug among the patients with hypoglycemic coma [27]. Quillam et al. have also proven an association between the use of TZD, sulphonylurea, and metformin and the development of hypoglycemia (33.3%, 42.3%, and 75.7%, respectively) [28].

The key motives of malnutrition in heart failure are neurohormonal changes and malabsorption. Hypercatabolism, which is activated by norepinephrine, adrenaline, and inflammatory cytokines, as well as reduced concentrations of anabolic hormones, resulted in malnutrition. One more cause of the malnutrition is altered intestinal function [29]. The narrowing of the vessels of the splanchnic circulation, provoked by elevated activity of the sympathetic nervous system, led to hypoxia and ischemia of the intestinal mucosa, which further led to a violation of the function of the epithelium and increased permeability of the mucous membrane [30].

There are many studies that have the same results. Patients suffering from heart failure had a lower BMI (21.9 ± 3.3 vs. 27.1 ± 4.4 kg/m^2^, *p* = 0.016) and a lower geriatric nutritional risk index (93.1 ± 13.3 vs. 117.6 ± 6.4, *p* < 0.01), a nutritional status indicator that was measured based on serum albumin and body weight, and a lower incidence of dyslipidemia (36.4% vs. 85.7%, *p* = 0.04), in contrast with patients without heart failure. The studies showed a violation of nutritional conditions in individuals with heart failure and heart failure-induced hypoglycemia, due to unhealthy diet [31].

It is also interesting to find out how it turned out that patients with developed hypoglycemia had no subjective symptoms. In patients with diabetes mellitus, the pathogenetic mechanism of silent hypoglycemia is familiar as vegetative insufficiency linked with hypoglycemia (HAAF) [32]. HAAF is a functional and reversible disorder that leads to impaired glucose regulation and ignorance of hypoglycemia caused by prior hypoglycemia. Earlier studies have reported that awareness of hypoglycemia decreases in patients with T1D and T2D mellitus with cardiac autoimmune neuropathy. Accordingly, hypoglycemia, which is provoked by malnutrition at an early stage of heart failure, can cause HAAF and result in asymptomatic hypoglycemia, including in patients without diabetes. In order to confirm this hypothesis, follow-up studies aimed at monitoring glucose levels in patients with heart failure with long-term follow-up are required [33,34].

However, hypoglycemia can be not only the consequence, but the cause of the heart failure [35].

In the Action to Control Cardiovascular Risk in Diabetes (ACCORD) trial, it was found that severe hypoglycemia episodes are linked to the increased cardiac troponin levels, which is a biomarker of myocardial injury, and echocardiographic signs of cardiac dysfunction [36].

So far, the pathogenesis and role of asymptomatic hypoglycemia in heart failure without glucose-lowering agents are not yet fully clear, but hypoglycemia may become a new therapeutic target in heart failure. In patients with increased cardiovascular risk and dysglycemia, acute hypoglycemia is linked with high risk of cardiovascular mortality. Thus, the treatment of hypoglycemia can result in an improvement in the prognosis for heart failure [37].

## 3. The Triglyceride-Glucose Index in CVD

Studies by Da Silva et al. demonstrated that the triglyceride-glucose index (TyG) was favorably linked with a higher incidence of symptomatic CAD [38]. In healthy people, TyG was used as a sign of insulin resistance. The application of this index as an identifier of atherosclerosis in patients with CVD may be influenced by diabetes and hyperlipidemic conditions that result in cardiovascular disease. Before claiming that the TyG index can be applied as a sign of atherosclerosis in patients with CVD, certain considerations may be required [39]. These factors can determine the role of fasting BG levels and triglycerides, which are used in the TyG formula. In order to demonstrate the value, the TyG index can bring to clinical practice, it is necessary to compare the levels of glucose and/or triglycerides in the fasting blood with the TyG index in these patients. The results of these studies may be unreliable due to these factors. Stratification by categories of CAD diseases does not contribute to understanding the role of TyG in the development of CVD. Correlation does not take into account the causation, so the use of the TyG index as an index in patients with coronary heart disease raises significant doubts [40].

In recent studies, the TyG index is actively used as an indicator of insulin resistance. It was reported that an increased TyG index is linked with an excessive risk of serious, negative cardiac and cerebrovascular events in patients with ST-segment elevation myocardial infarction (STEMI), who underwent percutaneous coronary intervention (PCI), and that the risk of ischemic stroke correlates with a proportional and linear growth in the TyG index [41]. Zhao et al. demonstrated that an elevated TyG index is mainly linked with an increased risk of arterial stiffness and damage to the microvessels of the kidneys. The TyG index is also used as a valuable biomarker of diabetes development, since it has demonstrated a linkage with the risk of diabetes [42].

Da Silva et al. examined individuals who had at least one CV event in the previous ten years; after that, those people were divided into several groups: (1) asymptomatic, (2) symptomatic, (3) and treated for CAD. Having calculated the TyG index in all these individuals, a statistically significant variance was found only in the group with symptoms (group 2), since a high TyG index had a higher incidence in patients with symptoms. This conclusion was confirmed by regression analysis of all groups. The outcomes were reliable, even when taking into account factors such as sex, age and the use of hypoglycemic, hypotensive, anticoagulant, and hypolipidemic agents [37]. It is important that all individuals who participated in the study were at risk of CAD, since they already had a history of CVDs. Diabetes is considered to be the key risk factor for CAD. Triglycerides are well-known, independent risk factors for cardiovascular diseases. Da Silva et al. did not provide any data on any statistics stratified by cardiovascular disease; therefore, most likely, many individuals in the symptomatic group had identical parameters regarding controlled factors (included in the regression model, especially the use of hypoglycemic, antihypertensive, anticoagulant, and hypolipidemic agents). As a result, control over these variables does not greatly affect the result.

The reason why symptomatic patients were treated with a higher percentage of the TyG index is that they had uncontrolled diabetes and/or hyperlipidemia, which resulted in increased levels of the TyG index, since TyG has a direct association with triglycerides and glucose (based on the TyG formula). This pattern was not observed in groups 1 and 3 (asymptomatic and treated groups), since they apparently controlled these factors (good treatment and lifestyle habits with asymptomatic and good treatment regimen and medications in the treated group) [43]. In the stated article, another point was missed. The authors could compare the diagnostic values of fasting glucose and triglyceride levels (and possibly combinations) with the TyG index, and then make an attempt to demonstrate that the TyG index may have a better diagnostic value than fasting glucose and triglyceride levels. In order to identify high-risk patients, especially patients with cardiovascular diseases, the medical doctor first checks the level of glucose and triglycerides on an empty stomach. How can the TyG index increase the predictive value of triglyceride and glucose levels? Since cardiovascular disease is a dynamic and progressive disease and the beginning of treatment should rely on the individual case of each patient, the use of indicators such as the TYG index as prognostic markers is less certain [44].

The use of the TyG index in individuals with cardiovascular disease can be easily overlooked, due to diabetes and hyperlipidemia, and these factors must be well controlled in order to explain its use as a biomarker. It should not be concluded that there is an inverse causality when using the Tug index in individuals with CVD [45].

## 4. LDL Level

Another important marker can be the level of LDL cholesterol and apolipoprotein B (apoB) 100, that is the major structural protein of LDL. These levels are directly associated with the risk for atherosclerotic cardiovascular events (ASCVE). The fact that the lowering of LDL-cholesterol by the use of statins decreases the cardiovascular events risk shows the importance of cholesterol for atherogenesis [46].

## 5. CVOTs and Data Overview

Over the past 20 years, robust cardiovascular outcome trials (CVOTs) have been conducted among patients suffering from T2D with a high risk of CVD developing. These studies became the most important stage that helped change the paradigm of treatment from a glucocentric approach to the treatment of diabetes to an approach in which the risk of cardiovascular diseases, heart failure, or CVD is taken into account when deciding on the best method of therapeutic intervention [47]. Since the 2008 mandate, no licensed antihyperglycemic drugs have demonstrated any problems with CV safety, in contrast to placebos. Therefore, not so long ago, the FDA rethought its guidelines. Now, pharmaceutical companies are not required to conduct special CVOTs to identify the CV safety of newer antihyperglycemic agents; on the other hand, it is assumed that pharmaceutical companies will continue to provide reliable evidence in registration studies of the general safety of the medication. CVOTS conducted to date are extremely important to help clinicians make individual decisions regarding their patients who suffer from diabetes mellitus and have a high risk of cardiovascular diseases. This has led to the use of therapeutic agents for the treatment of type 2 diabetes mellitus, which have cardiorenal benefits, regardless of the level of HbA1c [48] (see Table 1). 

Below, in Table 1, we have collected data on individual trials, including: ORIGIN trial, DEVOTE trial, RECORD trial, PROactive trial, IRIS trial, EXAMINE trial, SAVOR TIMI 53 trial, TECOS trial, CARMELINA trial, CAROLINA trial, LEADER trial, SUSTAIN-6 trial, HARMONY trial, REWIND trial, ELIXA trial, EXSCEL trial, PIONEER-6 trial, EMPA-REG OUTCOME trial, CANVAS trial, DECLARE-TIMI 58 trial, and VERTIS-CV trial.

These data are also confirmed by meta-analyses that were carried out on the basis of the above-mentioned trials.

The results of the meta-analysis caused concern about acute MI and deaths from cardiovascular diseases, so the Food and Drug Administration imposed significant restrictions on rosiglitazone, which were mostly lifted in 2013, after the drug was reassessed based on the results of the RECORD study. (Rosiglitazone was evaluated by cardiac outcomes and regulation of glycemia in diabetes) [52].

According to a meta-analysis of 26 studies (total number of participants: 19,645 patients), the effect of pioglitazone on the primary or secondary prevention of CVD in patients with T2D or at high risk of its development was evaluated. On the one hand, pioglitazone reduced the increase in 3-P-P by 20%, non-fatal MI by 20%, and non-fatal stroke by 19%. On the other hand, it led to an increase in the number of hospitalizations for heart failure by 34% [72].

Meta-analysis of combined data from SAVOR-TIMI 53, EXAMINE, TECOS, and CARMELINA demonstrated a neutral effect of DPP4 inhibitors on MI [odds ratio (OR): 1.01; *p* = 0.88; 95% CI: 0.92–1.10], stroke (OR: 0.99; 95% CI: 0.87–1.13; *p* = 0.88), combined endpoints of MI and stroke (OR: 1.00; 95% CI: 0.93–1.08; *p* = 0.97), and CV death (OR: 0.99; 95% CI: 0.91–1.09; *p* = 0.87). There was also no particular effect of DPP4 inhibition on hospitalization for heart failure (HR: 1.06; 95% CI: 0.96–1.18; *p* = 0.24).

Kristensen et al. reported unpublished data on hospitalization due to heart failure from HARMONY Outcomes in a meta-analysis. It was demonstrated that HARMONY Outcomes was the only GLP-1 RA study that revealed a significant decrease in the number of hospitalizations due to heart failure (*p* = 0.019). No other GLP 1RA showed any significant difference in hospitalization due to heart failure and unstable angina.

A meta-analysis of these seven cardiovascular outcomes involving 56,004 participants (including liraglutide, semaglutide, lixisenatide, albiglutide, exenatide, and dulaglutide), demonstrated a relative risk decrease of 12% at the primary outcome of 3-P MACE (HR 0.88, 95% CI 0.82–0.94; *p* < 0.001). It was also revealed that over 3.2 years, the number needed to treat (NNT) to prevent a single MACE event was 75 (95% CI 50–151). 

Finally, a meta-analysis of 46,969 patients who participated in clinical trials of results with SGLT2i proved consistent cardioprotective effects in this class. Treatment with SGLT2i inhibitors was linked with a decreased risk of MACE (HR 0.90; 95% CI 0.85–0.95), without significant heterogeneity. Moreover, the risk of hospitalization due to heart failure was identical in all trials (HR 0.68; 95% CI 0.61–0.76; I^2^ = 0.0%); on the contrary, significant heterogeneity in relation to cardiovascular death was observed between trials (HR 0.85; 95% CI 0.78–0.93; Q-statistical, *p* = 0.02; I^2^ = 64.3%) [73].

The results of conducted CVOTs show the decreased rate of adverse cardiovascular events among patients who received glucose-lowering drugs. However, it is not correct to suggest the direct relationship between these two factors. We can propose that the decrease of adverse cardiovascular event rates is mediated by other mechanisms involved in the action of glucose-lowering drugs. These mechanisms need to be investigated further. The results of numerous CVOTs show that, in general, the incidence of adverse cardiovascular events is lower in people taking glucose-lowering drugs. The results are practically the same, depending on the drug used. Accordingly, all of these data further suggest that higher glucose levels in both diabetics and non-diabetics are associated with a higher incidence of adverse cardiovascular events.

## 6. Conclusions

Without a doubt, glucose levels are not just numbers in the context of cardiovascular disease. Glucose metabolism is closely related to the cardiovascular system, and its violations logically provoke unpleasant consequences for the entire system. Although elevated glucose levels are not currently recognized as an independent risk factor for the development of cardiovascular disease, it is clear that further research on the relationships is, firstly, necessary, and secondly, will lead to a paradigm shift. A good illustration in favor of this judgment is the abundance of available studies in which the use of hypoglycemic drugs also reduced the incidence of adverse cardiovascular events. However, it is likely that this may be due to additional, not yet described, molecular mechanisms. Although the bulk of our review focuses on elevated glucose levels, hypoglycemia is also associated with disorders such as heart failure. We believe that the level of glucose can, if not become an independent prognostic criterion, then serve as the basis for such. In particular, glucose levels can be an effective supplement to triglyceride and LDL levels.

## Figures and Tables

**Table 1:** CVOTs and their results.

Trial	Drug	Subjects	Effect	References
ORIGIN	Insuline glargine	12,537 individuals with type 2 diabetes, impaired fasting glucose levels or impaired glucose tolerance, with additional risk factors for cardiovascular diseases (average baseline hemoglobin A1c level 6.5%)	No changes between the groups in either of the concomitant outcomes or in the individual components of the two coprimary outcomes. There were also no drastic changes in mortality or microvascular events	[49,50]
DEVOTE	Insuline glargine/Insulin degludec	7637 participants with an average baseline HbA1c level of 8.4%	Safety profile of insulin degludec for the cardiovascular system was comparable to the safety profile of insulin glargine U100. No cardinal differences were observed in the other prespecified CV results	[51]
RECORD	rosiglitazone	4447 participants with type 2 diabetes mellitus and an average baseline HbA1c level of 7.9%	MACE had no differences between test and control groups	[52]
PROactive	pioglitazone	5238 patients with type 2 diabetes, with extensive microvascular diseases	No significant differences between test and control groups (primary outcomes). However, during about three years of observation, an increase in the frequency of heart failure in the pioglitazone group compared with placebo was detected (11% vs. 8%, *p* < 0.0001).	[53]
IRIS	pioglitazone	Non-diabetic individuals with baseline HbA1c level 5.8%	Over 4.8 years of follow-up, there were considerably fewer cases of 3–P MACE when taking pioglitazone, in contrast to conventional treatment. Pioglitazone was able to considerably lower the incidence of DM2	[54]
EXAMINE	Alogliptin	5380 registered patients with type 2 diabetes following an acute coronary syndrome event	No important differences between the groups in both primary and cumulative CV outcomes. Secondary CV outcomes also did not show important differences between the test and placebo group.	[55]
SAVOR TIMI 53	Saxagliptin	16,490; the majority of participants already had cardiovascular diseases	No important differences between the groups in both primary and cumulative CV outcomes. Secondary CV outcomes also did not show important differences between the test and placebo group.The linkage of saxagliptin with noticeably elevated rates of hospitalization for heart failure compared to conventional medical care was found.	[56]
TECOS	Sitagliptin	14,671; the majority of participants already had cardiovascular diseases	No important differences between the groups in both primary and cumulative CV outcomes. Secondary CV outcomes also did not show important differences between the test and placebo groups.	[57]
CARMELINA	Linagliptin	6991 TDM2 patients	No important differences between test and control groups.	[58]
CAROLINA	Linagliptin/glimepiride	6033 TDM2 patients	Linagliptin was similar to glimepiride in all components of MAC	[59]
LEADER	liraglutide injection once a day	9341 patients with type 2 diabetes who were at high risk for cardiovascular disease	Noticeable reduction in the primary result of 3-P MACE, decrease in mortality from CVD, death from any cause, and MI	[60]
SUSTAIN-6	semaglutide for injection once a week	3297 patients with type 2 diabetes	Significantly fewer participants in the semaglutide group suffered a non-fatal stroke and revascularization of coronary or peripheral arteries	[61]
HARMONY	albiglutide injection once a week	9400 patients aged ≥40 years with T2DM, prior atherosclerotic CV disease, and suboptimal glycemic control	Albiglutide treatment led to a rather noticeable decrease in the frequency of 3P-MACE	[62]
REWIND	dulaglutide injection once a week	9901 type 2 diabetes patients who had either a previous cardiovascular event or cardiovascular risk factors	Decrease in the primary total result of 3P-MACE in the group receiving dulaglutide, compared to placebo	[63,64]
ELIXA	lixisenatide injection once a day	6068 participants with type 2 diabetes mellitus who experienced an acute coronary event within 180 days before recruitment	Lixisenatide demonstrated a neutral effect on all the primary and secondary endpoints	[65]
EXSCEL	exenatide injection once a week	14,752 participants	Exenatide showed a neutral effect on all secondary outcomes	[66]
PIONEER-6	daily oral semaglutide	3183 participants	Decrease in all-cause mortality and cardiovascular death	[67]
EMPA-REG OUTCOME	empagliflozin	7020 participants	Decreased mortality from all causes, CV mortality, hospitalization due to heart failure, and hospitalization due to insufficiency or death from CV causes (excluding fatal stroke)	[68]
CANVAS	canagliflozin	4330 participants	Marked decrease in 3P-MACE, canagliflozin reduced the number of hospitalizations due to heart failure	[69]
DECLARE-TIMI 58	dapagliflozin	17,160 participants	3P-MACE indicators had no significant discrepancies between dapagliflozin and the placebo; the frequency of hospitalization with heart failure was decreased with the use of dapagliflozin	[70]
VERTIS-CV	ertugliflozin	8246 patients	No noticeable effect of ertugliflozin in contrast to the placebo on 3P-MACE; fewer patients receiving ertugliflozin were hospitalized due to heart failure, in contrast to patients treated with placebo.	[71]

## Data Availability

Not applicable.
